# Isolation and Genotyping of *Acanthamoeba* Strains from Environmental Sources in Ahvaz City, Khuzestan Province, Southern Iran

**Published:** 2012

**Authors:** M Rahdar, M Niyyati, M Salehi, M Feghhi, M Makvandi, M Pourmehdi, S Farnia

**Affiliations:** 1 Cellular and Molecular Research Center, Tropical and Infectious Diseases Research Center & Department of Parasitology, School of Medicine, Ahvaz JundiShapur University of Medical Sciences, Ahvaz, Iran; 2 Department of Parasitology and Mycology, School of Medicine, Shahid Beheshti University of Medical Sciences, Tehran, Iran; 3 Department of Ophthalmology, Imam Khomeini Hospital, Ahvaz Jundishapur University of Medical Sciences, Ahvaz, Iran; 4 Department of Virology, School of Medicine, Ahvaz JundiShapur University of Medical Sciences, Ahvaz, Iran; 5 Department of Epidemiology and Statistical, School of Veterinary, Shahid Chamran University, Ahvaz, Iran; 6 Department of Parasitology and Mycology, School of Public Health, Tehran University of Medical Sciences, Tehran, Iran

**Keywords:** *Acanthamoeba* spp., Water, Soil, TYI-S-33, Iran

## Abstract

**Background:**

*Acanthamoeba* spp. are free-living amoebae commonly found in the environmental sources such as water, soil, and air. This ubiquitous amoeba is the causative agent of amoebic keratitis (AK). The objective of the present study was to investigate the presence of *Acanthamoeba* spp. in water and soil sources in Ahvaz City, Khuzestan Province, southern Iran.

**Methods:**

In general, 110 samples of water and soil were taken from different localities of Ahvaz including agricultural canals, rivers, and swimming pools. Filtration and cultivation were carried out on non-nutrient agar medium (NNA). Axenic cultivation was performed for all of positive isolates. PCR analysis was conducted on positive samples. Sequencing was done for 15 PCR products. Genotypes were identified by Blast search and homology analysis.

**Results:**

*Acanthamoeba* spp. was found in 43 (71.6%) of samples of water and 13 (26%) soil samples. Genotyping of 15 samples proved that *Acanthamoeba* belonged to T4 (86.6%), T2 (6.6%), and T5 (6.6%) genotypes.

**Conclusion:**

TYI-S-33 medium could be better than PYG medium for *Acanthamoeba* axenic culture.

## Introduction

*Acanthamoeba spp*. are an opportunistic amphizoic protozoa, commonly found in the environment. Researches showed that *Acanthamoeba* can be found in different environmental sources such as water, soil, sewage, and swimming pool (1–3). The taxonomy and classification of these protozoa are still under revision by the successful application of molecular techniques (4, 5). Evolutionary studies have led to the identification of at least 17 genotypes (T1–T17) based on rRNA gene sequencing. Among *Acanthamoeba* genotypes, genotype T4 is the most prevalent type causing disease in human. (6, 7). Indeed, *Acanthamoeba* spp. are an opportunistic causative agent of nasopharyngeal and skin infections. Also some strains can cause granulomatous amoebic encephalitis (GAE). Thus, several species of *Acanthamoeba* has different clinical sign with the potential to cause a corneal infection termed *Acanthamoeba* keratitis (AK) (1, 3, 8, 9). Amoebic keratitis (AK) infection can occur through use of the contaminated contact lenses with non-sterile water or through bathing or swimming in a contaminant water (1, 3). *Acanthamoeba* could also act as a carrier of fungi, viruses, and bacteria such as *Legionella pneumophila*, and *Mycobacterium avium* (10, 11).

In Iran, Rezaeian and Baghaei confirmed the first case of *Acanthamoeba* keratitis by culture method (12). Recently, AK is rising in Iran and the world (6, 9). The presence of *Acanthamoeba* in water, soil, dust, cow faeces, and swimming pool has been shown in Iran (13). *Acanthamoeba* T4 genotype was isolated from biofilms and dust sources from hospitals (14). Additionally, *Acanthamoeba* have been isolated from tap waters of the hospitals in Iran (15).

Since there was no information regarding the distribution of *Acanthamoeba* in Khuzestan Province, the main objective of the present research was to investigate the presence of *Acanthamoeba* genus in environmental sources of Ahvaz City, Khuzestan Province, southern Iran. Genotypes of some strains were also determined using molecular analysis.

## Materials and Methods

### Sampling

Totally, 110 samples of water and soil were collected from different localities of Ahvaz City including Kian Pars, Hamidieh, KutAbdollah, Kut-e SeyedSaleh, Karkheh and Karoon Rivers, Hasirabad, Jangieh, ZeytonKarmandi,and ZeytonKargari. Sixty water samples were taken from agricultural canals (12), rivers (8), tap water (12), river basins (10), swimming pools (4), and water pool in parks (14) and 50 soil samples were collected from the above mentioned regions. These samples were examined in the laboratory of Protozoology Unit, Department of Parasitology and Mycology, School of Public Health, Tehran University of Medical Sciences, Iran.

### Non- nutrient agar culture

Approximately 500 ml of water were filtered through 0.45 µm pore-size cellulose nitrate membranes (13). 100-200 g of soil samples were dissolved in sterile distilled water and filtered as described above. The filters were placed on 1.5% Non-nutrient agar (NNA) medium which was prepared with amoeba Page Saline. Amoeba Page Saline consists of 2.5 mM NaCl, 1 mM KH2PO4, 0.5 mM Na2HPO4, 40 m CaCl2-6H2O, and 20 m MgSO2. 7H2O. The final pH of this solution was adjusted to about 6.9 with KOH.

A small scrap was done at the side of the filter so that amoeba appears on the surface when it penetrates in agar. The NNA medium was incubated in room temperature. The culture medium was followed for two months.

### Cloning

A piece of agar was taken from NNA medium which contained less fungi and more amoeba for cloning and placed on fresh NNA medium. All plates were sealed, incubated at 30°C, and monitored daily for two months so that the amoebas grew and changed into cysts. The media were washed with sterile PBS (pH= 7.2); the surface and the beneath of agar was scraped with a blade then, *Acanthamoeba* cysts were collected and their sediments were taken by centrifuging.

### Axenic culture

The axenic culture of positive water and soil samples was conducted on PYG medium but the amoebae were not adopted to this medium; therefore, the other medium, TYI-S-33 medium, including 100 deionized or glass-distilled water, 0.1 g of potassium phosphate, dibasic, 0.06 g of potassium phosphate, monobasic, 0.2 g of sodium chloride, 0.2 g of casein digest peptone, 2 g of yeast extract, 1 g of glucose, 0.1 g of L-cysteine hydrochloride, 0.1 g of ascorbic acid, and 0.0023 ml of ferric ammonium citrate was used for axenification (16).

### DNA extraction and PCR amplification assay

DNA from the positive samples was extracted by using the phenol–chloroform method as previously described (17). For extracting, DNA lysis buffer (50 mMNaCl, 10 mM EDTA, 50 mMTris–HCl, pH 8.0) and proteinase K (0.25 mg/ml) were used and incubated at 56 °C, for overnight.

PCR analysis was performed using JDP primers including: JDP1 forward 5'-GGCCCAGAT CGTTTACCGTGAA-3' and JDP2 reverse 5'-TCTCACAAGCTGCTAGGGGAGTCA-3'.

These primers approximately amplified a 500 bp fragment. PCR reaction was performed in 30 μl Ampliqone (Taq DNA Polymerase Master Mix RED, Denmark). Twenty-five microliters of Taq Master mix were used with 10 ng template DNA, 0.1 μM of each primer, and distilled water.

Cycles of PCR were set up as following: pre-denaturation step at 94 °C for 3 min and 33 cycles of denaturation at 95 °C for 35 S, annealing at56 °C for 45 S and extension at 72 °C for 1 min with an elongation step of 5 min at 72 °C at the last cycle.

### Gel electrophoresis

The PCR-products electrophoresis was done on 2% (w/v) agarose gel, stained with ethidium bromide solution (0.5 μg/ml) and visualized under UV light.

### Sequencing and genotyping of the isolates

PCR products of 15 isolates were purified using the Column-based Purification kit and sequenced using ABI 3730XL automatic sequencer by Fazabiotech Company. The obtained sequences were edited and aligned using Chromas software program. Genotype identification was done by comparing with available *Acanthamoeba* DNA sequences in the GenBank based on sequence analysis of DF3 region.

## Result

Fifty six out of 110 samples were positive for *Acanthamoeba* spp. based on morphology characterization. *Acanthamoeba* spp. were found in 43 (71.6%) water and 13 (26%) soil samples. The axenic culture on PYG medium was not successful but axenification on TYI-S-33 medium showed a considerable growth of the amoebae in a short time.

**Fig. 1:**
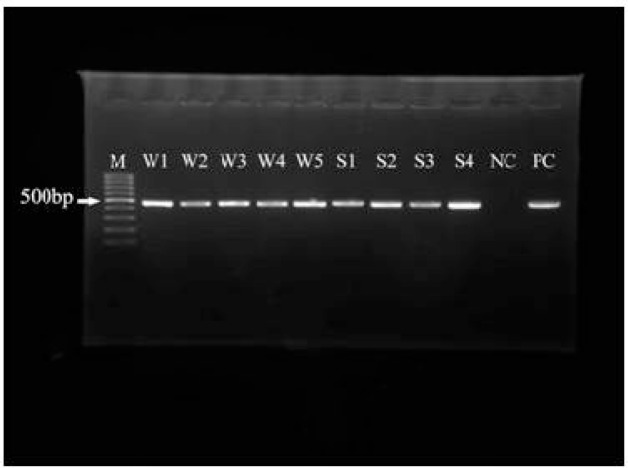
PCR-product of water and soil samples. M=Molecular weight marker (100bp), W=Water, S=Soil, NC=Negative Control, PC=Positive Control

PCR analysis of the positive samples using JDP1 and JDP2 was performed and a specific 500 bp band was detected on agarose gel for all positive isolates (Fig. 1).

The results obtained from sequencing of 9 water and 6 soil samples showed that genotypes T2 (6.6%) and T5 (6.6%) were related to water and soil isolates respectively and 13 (86.6%) remaining genotypes belonged to T4. Table 1 shows the genotypes and accession numbers of samples.

**Table 1:** Genotypes & accession numbers of water and soil samples

Sample ID	Genotype	Accession No.
aAw_1_	T4	JQ945977
a Aw_2_	T2	JQ945987
bAw_3_	T4	JQ945979
b Aw_4_	T4	JQ945976
c Aw_5_	T4	JQ945981
c Aw_6_	T4	JQ945982
dAw_7_	T4	JQ945983
e Aw_8_	T4	JQ945986
f Aw_9_	T4	JQ945978
As_1_	T5	JQ945980
As_2_	T4	JQ945984
As_3_	T4	JQ945985
As_4_	T4	JQ945988
As_5_	T4	JQ945989
As_6_	T4	JQ945990

Aw Ahvaz water/As Ahvaz soil

a Agricultural canals

b Water of pools(in park)

c River basins

d Karoon rivers

e Karkheh River

f Tap water

## Discussion

This study indicated the present of *Acanthamoeba* spp. in water and soil samples in Ahvaz City. Human activity was seen in all of the localities from which samples were taken. 71.6% of the flowed water in agricultural canals, tap water, pool water, river basins, and rivers were contaminated with *Acanthamoeba* spp. However, 26% of the soil samples were contaminated with amoebae. These findings indicate that less soil samples were contaminated with *Acanthamoeba* spp. in comparison with waters. A previous study in Iran, have proved the presence of *Acanthamoeba* spp. in different environmental sources such as water, soil, dust, and cow faeces (18). *Acanthamoeba* spp. has also been isolated from drinking waters of several hospitals in Iran; the prevalence of *Acanthamoeba* in hospitals of Ahvaz city was reported as 50% (15). This finding showed the risk of being affected by *Acanthamoeba* at hospitals. *Acanthamoeba* spp. were also isolated from the biofilm in Tehran hospitals which immune deficiency patients were hospitalized and the most of their genotypes belonged to T4 (14). High percentage of *Acanthamoeba* spp. in water, soil, and other environmental samples is a hygienic risk for public health especially for individuals with immune deficiency situation and contact lens wearers (19). Besides, *Acanthamoeba* spp. have been isolated from keratitis patients using molecular method in Iran that belonged to T4 genotype (6).

PCR analysis and sequencing of isolates in this study revealed the existence of T4,T5, T2 genotypes in water and soil of this region. The most frequency genotype isolated were T4 genotype which agree with other researches in Iran. Indeed, the presence of *Acanthamoeba* spp. in water where human activity is high may cause the infection in contact lens wearers (20). The high rate of *Acanthamoeba* spp. presence in water of different localities of Ahvaz indicated the high rate of contamination of this environment with this free-living amoeba and T4 genotype. The existence of *Acanthamoeba* cyst in soil indicated its resistance to high temperature of this region. In the present study, *Acanthamoeba* was isolated from tap water and agriculture canals which are a hazard for the people who drink and use this water in their routine life. Also, swimming in pools can also threaten the health conditions of children.

The most important findings of this study were the existence of T4 genotype in this region and introducing TYI-S-33 medium for *Acanthamoeba* axenic culture. The axenic culture in PYG medium needs high rate of *Acanthamoeba* cyst; moreover, growth in this medium takes a long time which provides the appropriate conditions for bacteria and fungi growth. Although, the growth in this medium was not successful, culturing in TYI-S-33 medium needed very small rate of cyst and amoeba in this medium grew very rapidly which can prevent the growth of acteria and fungi. Therefore, this medium can substitute PYG medium.

## Conclusion

While *Acanthamoeba* spp. are free living amoebae in our surrounding environment and we are exposed to them every day without being aware therefore, considering health principles are suggested.
